# Ubiquitin-specific protease 3 promotes cell migration and invasion by interacting with and deubiquitinating SUZ12 in gastric cancer

**DOI:** 10.1186/s13046-019-1270-4

**Published:** 2019-06-24

**Authors:** Xiaosheng Wu, Mengwei Liu, Huiqiong Zhu, Jing Wang, Weiyu Dai, Jiaying Li, Danping Zhu, Weimei Tang, Yizhi Xiao, Jianjiao Lin, Wenjing Zhang, Yong Sun, Yi Zhang, Yaying Chen, Guoxin Li, Aimin Li, Li Xiang, Side Liu, Jide Wang

**Affiliations:** 1 Guangdong Provincial Key Laboratory of Gastroenterology, Department of Gastroenterology, Nanfang Hospital, Southern Medical University, Guangzhou, 510515 China; 2 Department of Clinical Laboratory, General Hospital of Southern Theatre Command, Guangzhou, 510010 China; 3 Department of Gastroenterology, Longgang District People’s Hospital, Shenzhen, 518172 China; 4 Department of Medical Oncology, the First people’s Hospital of Yunnan Province, Medical School of Kunming University of Science and Technology, Kunming, 650032 China; 5 Department of Gastroenterology, The third affiliated Hospital of Guangzhou Medical University, Guangzhou, 510150 China; 6 Department of General Surgery, Nanfang Hospital, Southern Medical University, Guangzhou, 510515 China; 7 Department of Digestive Medicine, Nanfang Hospital, Southern Medical University, Guangzhou, 510515 Guangdong Province China

**Keywords:** USP3, SUZ12, Gastric cancer, EMT, iTRAQ

## Abstract

**Background:**

The deubiquitinating enzyme ubiquitin-specific protease 3 (USP3) plays a crucial role in numerous biological processes. The aberrant expression of USP3 may have an important role in tumor development. However, the mechanism by which USP3 promotes gastric cancer (GC) metastasis remains largely unknown.

**Methods:**

Effects of USP3 on the progression of GC in vivo and in vitro and the potential underlying mechanisms have been investigated utilizing proteomics, RT-PCR, western blotting, immunohistochemistry, immunofluorescence, cell invasion and migration assays and xenograft tumor models.

**Results:**

USP3 expression was upregulated in GC compared with matched normal tissues and was predictive of poor survival. USP3 also promoted migration and epithelial-to-mesenchymal transition (EMT) in GC cells. Moreover, TGF-β1 induced USP3 expression, and USP3 knockdown inhibited TGF-β1-induced EMT. Furthermore, we utilized Isobaric Tag for Relative and Absolute Quantitation (iTRAQ) to identify differentially expressed proteins in USP3-overexpressing cells compared with control cells. Importantly, we found that SUZ12 is indispensable for USP3-mediated oncogenic activity in GC. We observed that USP3 interacted with and stabilized SUZ12 via deubiquitination. SUZ12 knockdown inhibited USP3-induced migration and invasion, as well as EMT in GC cells. Examination of clinical samples confirmed that USP3 expression was positively correlated with SUZ12 protein expression and that the levels of USP3 or SUZ12 protein were negatively correlated with the levels of E-cadherin protein.

**Conclusions:**

These findings identify USP3 as a critical regulator. The USP3-SUZ12 axis might promote tumor progression and could be a potential therapeutic candidate for human GC.

**Electronic supplementary material:**

The online version of this article (10.1186/s13046-019-1270-4) contains supplementary material, which is available to authorized users.

## Background

Gastric cancer (GC) is the fifth most common malignancy and the third leading cause of cancer-related deaths worldwide [1]. GC metastasis, which is responsible for almost 90% of the lethality among GC patients, is a multistep process that results from alterations in genes, including the activation of oncogenes and the inactivation and mutation of tumor suppressor genes [2]. Through greater awareness, earlier detection, and advances in treatment, the survival of GC patients has improved; however, a proportion of patients will eventually succumb to the disease as a result of metastasis [3]. Therefore, the identification of novel genes and the discovery of the detailed mechanisms underlying GC metastasis are urgently needed.

The ubiquitin-proteasome pathway is an important intracellular protein degradation regulatory system [4]. Deubiquitination is an important opposing mechanism of ubiquitination and is catalyzed by deubiquitinating enzymes (DUBs). Approximately 100 DUBs have been reported to be encoded by human genes and are divided into 6 subclasses based on their Ub-protease domains [5, 6]. The ubiquitin-specific proteases (USPs) are the largest subclass of DUBs [7]. The USP3 gene is a member of the USP family. Recent progress has highlighted the significance of USP3 in tumor progression. For example, in one study, USP3 was overexpressed in GC tissues and cells, and ectopic USP3 promoted GC tumor growth [8]. USP3 participates in the reverse process of ubiquitination by removing ubiquitin from target proteins and rescuing proteins that are marked for degradation [9]. USP3 was reported to deubiquitinate RIG-1-like receptors and to inhibit type I interferon signaling [10]. However, it is unclear whether USP3 modulates target proteins to regulate cell migration and invasion in GC.

SUZ12 (Suppressor of zeste 12 protein homolog) is a core component of the Polycomb PRC2-HMTase complex, which has been shown to be involved in stem cell maintenance and development [11, 12]. SUZ12 is upregulated in many human cancers including colon [13], liver [14], gastric [15] and breast cancers [16]. For example, SUZ12 expression was significantly increased in gastric tumor tissues compared with normal tissues [15]. SUZ12 expression was found to be associated with pathological stage, metastasis distance, and shorter overall survival of GC patients. Moreover, SUZ12 induced tumor cell epithelial-to-mesenchymal transition (EMT) and maintained the invasive potential of cancer, which appeared to play a crucial role in the metastatic progression of human carcinomas [16, 17].

Based on these findings, we further investigated the molecular mechanism by which USP3 regulates GC progression. In this study, we found that TGF-β-induced USP3 promotes EMT and metastasis. We also demonstrated that USP3 is a positive regulator of SUZ12 and that it deubiquitinates and stabilizes SUZ12 and promotes progression of GC.

## Materials and methods

### Cells culture, antibodies and reagents

See Additional file 1: Supplementary materials and methods.

### **Tissue** multi-**array** (**TMA**) and immunohistochemistry

The GC samples and the corresponding adjacent nontumor tissues were used to construct a tissue microarray (TMA; Shanghai Biochip Co., Ltd., Shanghai, China). The tissue microarray was stained for USP3 expression. The TMA was scored independently by two pathologists for both staining intensity and extent of protein expression across the section. Immunohistochemical staining was performed using the Dako Envision Plus System (Dako, Carpinteria, CA, USA) according to the manufacturer’s instructions. Analysis was performed by two independent observers who were blinded to the clinical outcome. Staining intensity was scored as 0, negative staining; 1, weak staining; 2, moderate staining; and 3, intense staining. Cells with negative or weak staining were defined as low expressers, and cells with moderate or intense staining were defined as high expressers, as previously described [18]. This study was approved by the institutional human ethics committee of the relevant institutions.

### Plasmid construction and small interfering RNA transfection

The USP3 or SUZ12 coding sequence (CDS) was cloned into the pENTER shuttle plasmid (ViGene Biosciences Inc., Rockville, MD, USA). pENTER- USP3 and SUZ12 and empty vector (pENTER) plasmids encoding FLAG or MYC tag were purchased from Vigene. Populations of pENTER vector and pENTER USP3 or SUZ12 pooled stable transfectants of GC lines were obtained using the same plasmid and selection process as described previously [19].

For RNA interference studies, GC cells were transfected with predesigned small interfering RNAs (siRNAs) at final concentrations of 25 nM. The siRNA sequences were as follows: USP3 sense strand: siRNA 1: TTCACAGCTGACAGGCATA, siRNA 2: CCTTCAGTCACTCAGTAAC, siRNA 3: CCATGAATTCATGCGCTAC; SUZ12 sense strand: siRNA 1: GTCTCATCGAAACTCCAGA. Src siRNA, 5′-TTCTCCGAACGTGTCACGT-3′, which did not target any genes, was used as the negative control. The cells were transfected with siRNA duplexes using oligofectamine (Invitrogen-Life Technologies) according to the manufacturer’s instructions. The effect of gene knockdown was evaluated by western blotting 48 h post transfection.

### Co-immunoprecipitations and Western blotting analysis

See Additional file 1: Supplementary materials and methods.

### Quantitative RT-qPCR

See Additional file 1: Supplementary materials and methods.

### Wound-healing assays

See Additional file 1: Supplementary materials and methods.

### Invasion assays

See Additional file 1: Supplementary materials and methods.

### Immunofluorescence staining and confocal laser scanning microscopy

See Additional file 1: Supplementary materials and methods.

### Protein extraction and preparation for isobaric tag for relative and absolute quantitation (iTRAQ)

The BGC-823 cells were lysed in lysis buffer (20 mM Tris–HCl, pH 7.5, 150 mM NaCl, 1 mM EDTA, and 1% Triton X-100 with protease inhibitor) and sonicated, and the supernatant was collected by centrifugation at 30,000 g for 15 min. After treatment with acetone, the cell extracts were resuspended in 250 mM triethylammonium bicarbonate (TEAB), which was followed by incubation with 10 mM DL-dithiothreitol (DTT) in a water bath at 56 °C for 1 h, and then with 55 mM iodoacetamide (IAM) in the dark at room temperature for 45 min. The proteins (100 μg each) were digested in trypsin at 37 °C for 16 h. Subsequently, the peptides were dried by vacuum centrifuge and resuspended in 500 mM TEAB for further iTRAQ labeling. The protein concentration was determined using a Bradford assay.

### iTRAQ labeling and strong cation exchange (SCX) separation

The iTRAQ labeling of tryptic peptides was performed using an iTRAQ reagent Multiplex kit (Applied Biosystems, Foster City, CA, USA) according to the manufacturer’s protocol. The peptides labeled with respective isobaric tags were incubated at room temperature for 2 h and vacuum centrifuged until dry. Then, the iTRAQ-labeled peptide samples were reconstituted in Buffer A [25 mM NaH_2_PO_4_, 25% acetonitrile (ACN), pH 2.7] and fractionated using an Ultremex SCX column (250 × 4.6 mm, 5 μm particle size, 200 Å pore size) via an LC-20AB HPLC pump system (Shimadzu, Japan) at a flow rate of 1.0 ml/min. The 35 min HPLC gradient consisted of 100% Buffer A for 10 min, 5–35% Buffer B (25 mM NaH_2_PO_4_, 25% ACN, 1 M KCl, pH 2.7) for 11 min, 35–80% Buffer B for 1 min, 80% Buffer B for 3 min and 100% Buffer A for 10 min. The chromatograms were recorded at 214 nm. The collected fractions were desalted using a Strata X C18 column (Phenomenex), vacuum centrifuged and reconstituted in 0.1% formic acid (FA) for subsequent LC–MS/MS analysis.

### Mass spectrometric analysis

Samples were analyzed on a Thermo Scientific Q Exactive mass spectrometer (Fitgene Biotechnology CO., LTD, Guangzhou, China). The peptides were subjected to nanoelectrospray ionization (NSI) followed by tandem mass spectrometry (MS/MS) in a QEXACTIVE (Thermo Fisher Scientific, San Jose, CA) coupled online to the HPLC. Protein identification was performed with MASCOT software by searching UniProt_Aedis. Differentially expressed proteins were filtered for a fold change cutoff of 1.2 and a *p*-value cutoff of 0.05. The experiments were repeated three times.

### Cycloheximide treatment

To determine the stability of endogenous SUZ12 protein, cells were transfected with either empty plasmid or the pENTER-FLAG-USP3 plasmid with 100 μM cycloheximide for the times indicated in the figure legends. Total protein amounts of cell lysates were determined by BCA assay. Lysates with equivalent protein amounts were resolved by SDS-PAGE and analyzed by Western blotting to determine the abundance of endogenous SUZ12 protein.

### In vitro deubiquitylation assay

Deubiquitination assays were conducted as previously described [9, 10]. In brief, for analyzing the ubiquitination of SUZ12, BCG-823 cells was transfected with Flag-USP3 or vector for 48 h. Cell lysates were prepared with cell lysis buffer. Whole-cell lysates were immunoprecipitated with the indicated antibodies (anti-SUZ12 or IgG) on protein A/G beads (Santa Cruz Biotechnology) overnight. The beads were then washed and boiled in SDS loading buffer. Immunoprecipitated protein complexes ((anti-SUZ12, anti-USP3, anti-poly-UBC and anti-GAPDH) were assessed using Western blotting.

### Protein degradation assay

MG132 (carbobenzoxy-Leu-Leu-leucinal) was purchased from Sigma-Aldrich. MG132 is a peptide aldehyde, which effectively blocks the proteolytic activity of the 26S proteasome complex. We measured the SUZ12 expression level following treatment with MG132 under different USP3 conditions to confirm whether USP3 inhibits SUZ12 degradation.

### Construction of lentivirus and orthotopic mouse metastatic model

Lentivirus expressing EGFP/USP3 (LV- USP3) was constructed by Genechem (Shanghai, China) using Ubi-MCS-3FLAG-CBh-gcGFP-IRES-puromycin vector. Ubi-MCS-3FLAG-CBh-gcGFP-IRES-puromycin empty vector were used as controls. Selected pools of over expressing cells were used for subsequent experiments.

A surgical orthotopic implantation mouse model of GC was performed as described previously [20]. A single-cell suspension of 5 × 10^6^ BCG-823/pEGFP/vector and BCG-823/EGFP/USP3 -transduced cells/100 μl PBS was injected via the tail vein. The progression of cancer cell growth was monitored after 34 days by bioluminescent imaging using the IVIS100 Imaging System (Kodak, Rochester, NY, USA). Before surgical and analytical procedures were performed, the mice were anesthetized. The metastatic tissues were analyzed by hematoxylin and eosin staining, immunohistochemistry (IHC) and qRT-PCR. The Committee on the Use of Live Animals in Teaching and Research, the Southern Medical University,China, approved the protocol (authorization protocol number: NFEC-2017-062).

### Statistical analysis

Statistical analyses were performed using the SPSS statistical software package (standard version 20.0 PSS, Chicago, IL). Quantitative data obtained from experiments with biological replicates are shown as the mean ± standard deviation. Linear regression and Pearson correlation analysis were performed. Survival analysis was performed with the Kaplan-Meier and log-rank tests. Two-tailed Student’s t-test was used to analyze the quantitative data with significant difference being considered if *P* values was < 0.05.

## Results

### Increased USP3 expression is correlated with tumor progression and poor prognosis in GC patients

**Fig. 1:**
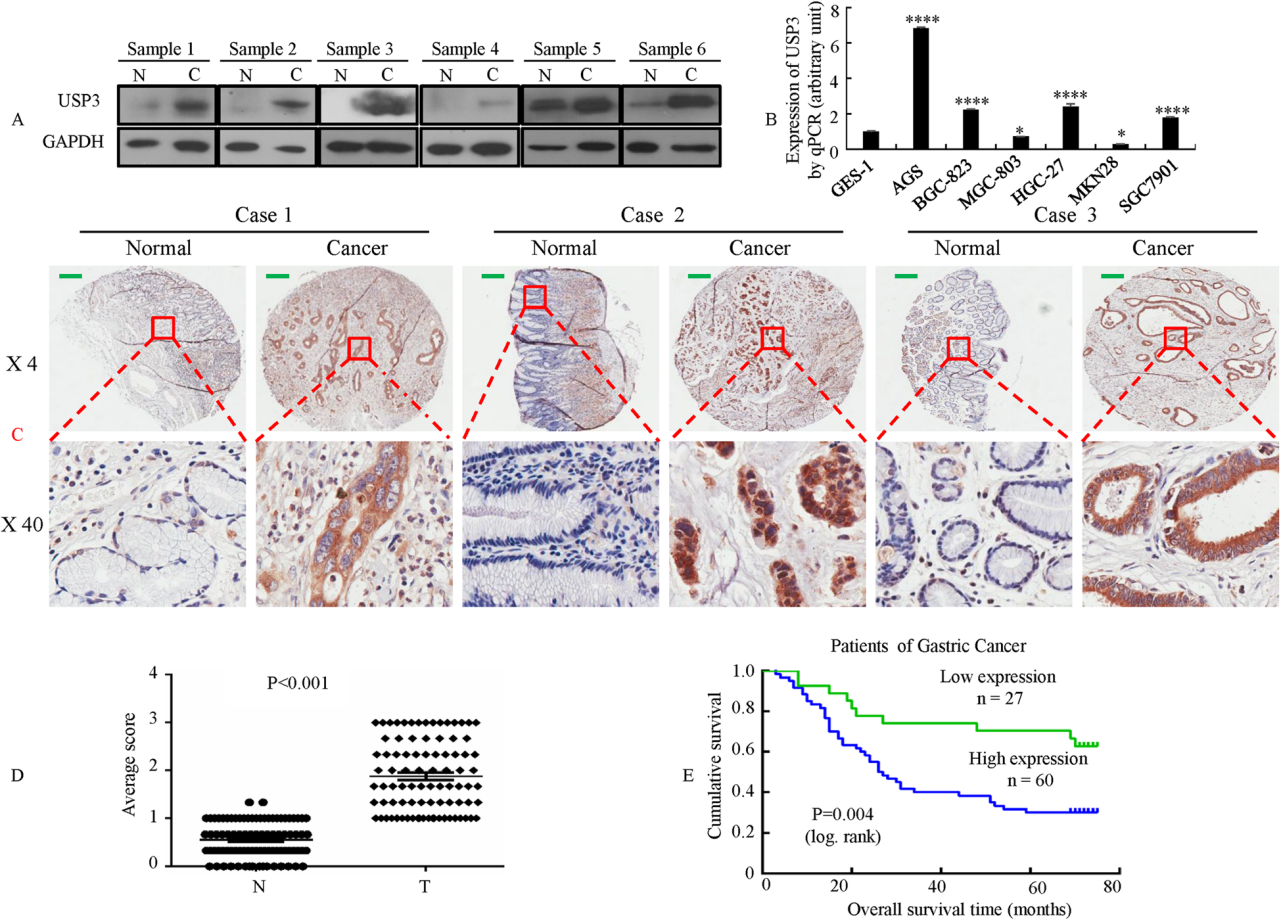
USP3 expression in gastric cancer (GC) was associated with a poor prognosis. **a** Western blot analysis of USP3 levels in human GC tissues and adjacent nontumor tissues. Expression levels of USP3 were normalized to the expression level of GAPDH. **b** The expression of USP3 mRNA in immortalised gastric mucosal cell line GES-1 and gastric cancer cell lines AGS, BGC-823, MGC-803, HGC-27, MKN28 and SGC7901 as detected via quantitative real-time RT-PCR. The experiment was performed intriplicate. *, *p* > 0.05; ****, *p* < 0.001. **c** USP3 expression in 87 samples of either normal or cancerous gastric tissues was detected by TMA analysis. **d** As analyzed by IHC, the USP3 expression level in GC tissues was significantly higher than that observed in the corresponding adjacent nontumor tissues. **** *p* < 0.001. **e** Kaplan-Meier survival analysis of overall survival in all patients (**e**) according to USP3 expression. The log-rank test was used to calculate *p* values. Scale bars, 200 μm in C

To determine the role of USP3 in the aggressiveness of GC, we investigated the expression levels of USP3 by Western blot in 6 pairs of GC tissues. The results showed that USP3 protein was upregulated in GC tissues compared with adjacent nontumor tissues (Fig. 1a). Next, the expression of USP3 was detected in six cancer cell lines by semiquantitative RT-PCR assay. As shown in Fig. 1b, the GC cell lines AGS, BGC-823, HGC-27 and SGC-7901 showed elevated expression of USP3, while MGC-803 and MKN28 did not demonstrate increased USP3 expression levels compared with human gastric epithelial cell line GES-1.

Moreover, USP3 expression was analyzed in 87 GC tissue samples and was compared with the expression in adjacent nontumor tissues by tissue microarray (TMA). The human GC tissues exhibited greater immunostaining, whereas the normal gastric tissues exhibited less immunostaining (Fig. 1c). Semiquantitative scoring showed that USP3 protein was expressed at significantly higher levels in cancer tissues compared with adjacent nontumor tissues (Fig. 1d).

Clinicopathologic analysis revealed that expression of USP3 was positively correlated with tumor differentiation status (*P* < 0.001), lymph node metastasis (*P* = 0.013), tumor size (< 10 cm vs ≥ 10 cm, *P* = 0.016), AJCC T stage (I/II vs. III/IV, *P* = 0.029), and clinical TNM stage (I/II vs. III/IV, P < 0.001). USP3 staining did not significantly correlate with age (*P* = 0.383) or gender (*P* = 0.808) (Additional file 1: Table S1).

The overall survival rate of GC patients with high USP3 expression was significantly poorer than that of patients with low USP3 expression by the Kaplan-Meier method (*P* = 0.004; Fig. 1e).

Collectively, these results suggested that USP3 may play a role in GC development and progression.

### Upregulation of USP3 promotes metastasis through EMT in GC

**Fig. 2:**
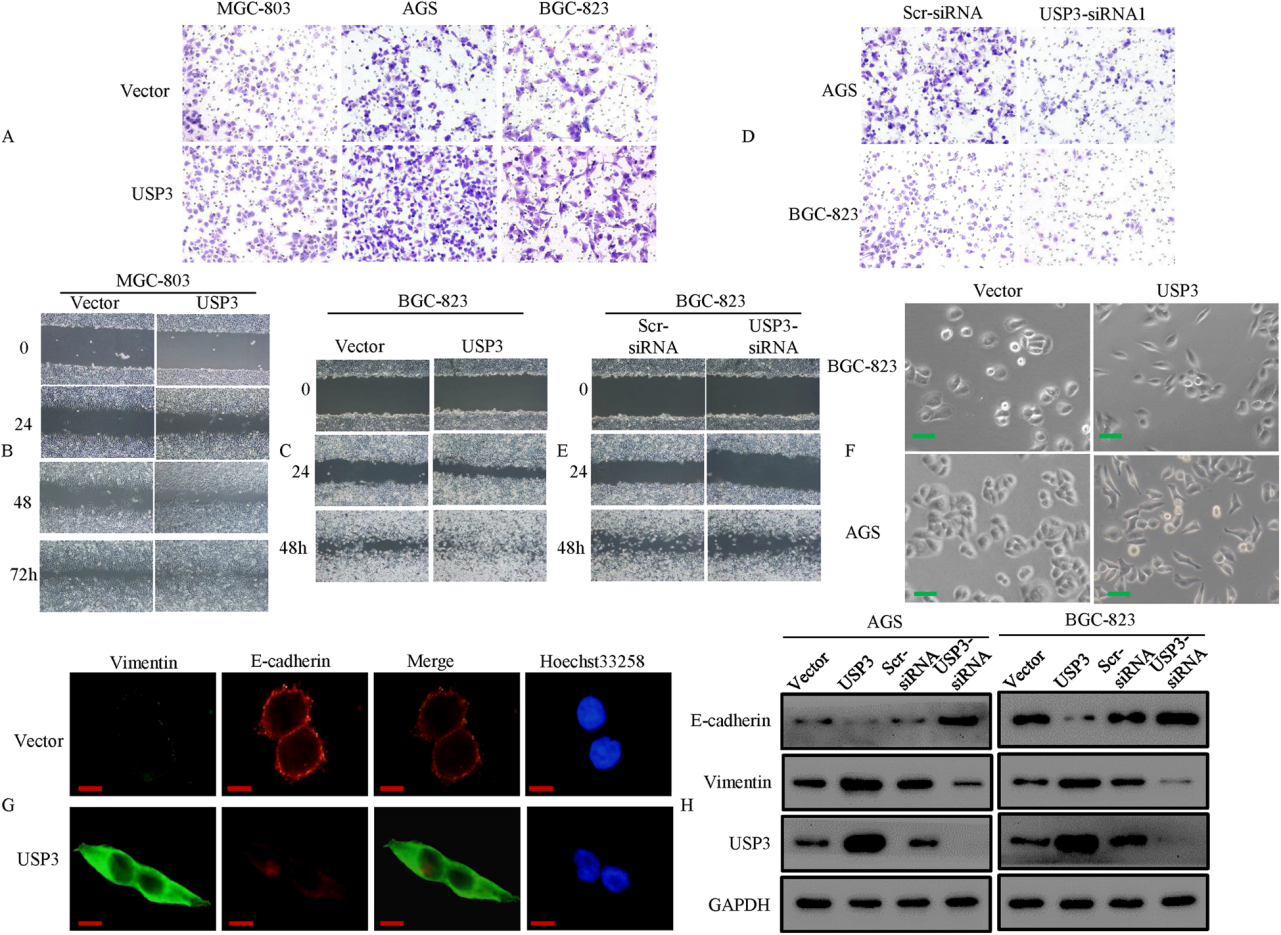
Overexpression of USP3 promotes the invasive and metastatic abilities of GC cells. **a** Comparison of the invasion potential of GC cells transfected with vector and USP3. **b** & (**c**) Representative images of the wound-healing assay in MGC-803 and BGC-823 cells. **d** & (**e**) The effect of RNA interference (RNAi) on USP3 gene mRNA expression and the invasive and migration potential of human GC cell lines. **f** Morphology of pooled cells stably transfected with vector or USP3 as visualized by phase-contrast microscopy. **g** E-cadherin and Vimentin expression was detected by cell immunofluorescence in BGC-823 cells. **h** Expression of epithelial markers and mesenchymal markers in vector- or USP3-transfected cells was assessed by Western blot. GAPDH was used as a loading control. Scale bars represent 50 μm in (**f**) and 20 μm in (**g**)

Elevated cell migration and invasion are associated with the increased metastatic potential of cancer cells [21, 22], which may be independent of cell proliferation rates. Therefore, we studied the effect of USP3 on cell invasion and migration of MGC-803 (Low-level expression, Fig. 1b) and AGS and BGC-823 (High-level expression, Fig. 1b) cell lines using the transwell and wound-healing assay. The data showed that ectopic expression of USP3 promoted GC cells invasion and migration compared with the vector control cells (Fig. 2a-c). Moreover, the AGS and BGC-823 cells showed higher invasion and migration rates compared to the MGC-803 cells (Fig. 2a-c, Additional file 2: Figure S1A-C). Then, we synthesized 3 pairs of USP3 siRNA (pool siRNA oligonucleotides). We showed that knock-down of USP3 could inhibit the invasive and migration abilities of AGS and BGC-823 cells (Fig. 2d & e; Additional file 2: Figure S1D & E). These results suggest that high-level expression of USP3 may contribute to the metastasis of GC by promoting the invasion and migration ability of GC cells.

The acquisition of an EMT phenotype is a critical process for switching early stage carcinomas into invasive malignancies, which is often associated with the loss of epithelial differentiation and gain of mesenchymal phenotype [20, 23]. We next examined the morphologic features of GC cells. The stable vector-transfected AGS and BGC-823 cells exhibited a cobblestone-like typical epithelial morphology and were present as a confluent monolayer or as islands of grouped cells with tight cell-cell contacts. However, the USP3-transfected cells underwent a striking morphologic change, which was characterized by loss of cell-cell contacts and a cobblestone-like phenotype, failure to form a confluent monolayer or islands, and the gain of an elongated and spindle-shaped morphology and scattered appearance. These phenotypes are typical of the fibroblastoid cells generated during the EMT process (Fig. 2f).

Expression of epithelial and mesenchymal molecular markers was confirmed by immunofluorescence and western blot. Expression of epithelial markers, E-cadherin, was significantly reduced in USP3 transfectants cells. In contrast, expression of mesenchymal markers, vimentin (which positively correlate with EMT), was dramatically upregulated in USP3 transfectants cells, and vice versa (Fig. 2g & h).

Several studies have shown that phosphorylation level proteins which involved ERKl/2/Akt signaling has emerged as a central feature of EMT, which is the initial step for metastasis in many cancer models [19, 22]. We performed western blot analysis to elucidate the phosphorylation status of proteins. We showed that the phosphorylation levels of AKT and ERK1/2 increased in USP3-overexpressing cells; while the level of p-AKT and ERK1/2 decreased siRNA-mediated knockdown of USP3 in GC cells. The total amount of AKT and ERK1/2 protein was unaltered (Additional file 2: Figure S1F). Moreover, the expression level of the typical EMT epithelial marker E-cadherin was downregulated in USP3-overexpressing cells and vice versa (Additional file 2: Figure S1F).

Taken together, these observations suggest that the overexpression of USP3 facilitates gastric cancer invasion and metastasis through inducing EMT program.

### USP3 is required for TGF-β1-induced EMT and cell migration in GC

Several studies have reported that TGF-β induces EMT in GC cells [24, 25]. To investigate whether TGF-β affects USP3 expression, we first examined the levels of USP3 in TGF-β-treated BCG-823 cells. Notably, TGF-β treatment resulted in a significant increase in USP3 expression in a dose- and time-dependent manner. Moreover, TGF-β1 markedly inhibited the expression of the EMT marker E-cadherin (Additional file 3: Figure S2A & B). Blocking TGF-β1 with a neutralizing antibody significantly suppressed endogenous USP3 expression and TGF-β1-induced USP3 expression in BGC-823 cells (Additional file 3: Figure S2C). However, pretransfecting cells with USP3 siRNA not only inhibited USP3 expression but also suppressed TGF-β1-induced vimentin expression (Additional file 3: Figure S2D). In addition, the results indicated that USP3 siRNA neutralized the influence of TGF-β1 on cell phenotypes (Additional file 3: Figure S2E). Furthermore, downregulation of USP3 significantly antagonized the TGF-β-induced invasiveness of GC cells (Additional file 3: Figure S2F).

These experiments suggest that USP3 is involved in and required for TGF-β-induced EMT.

### USP3 promotes EMT and metastasis in vivo

**Fig. 3:**
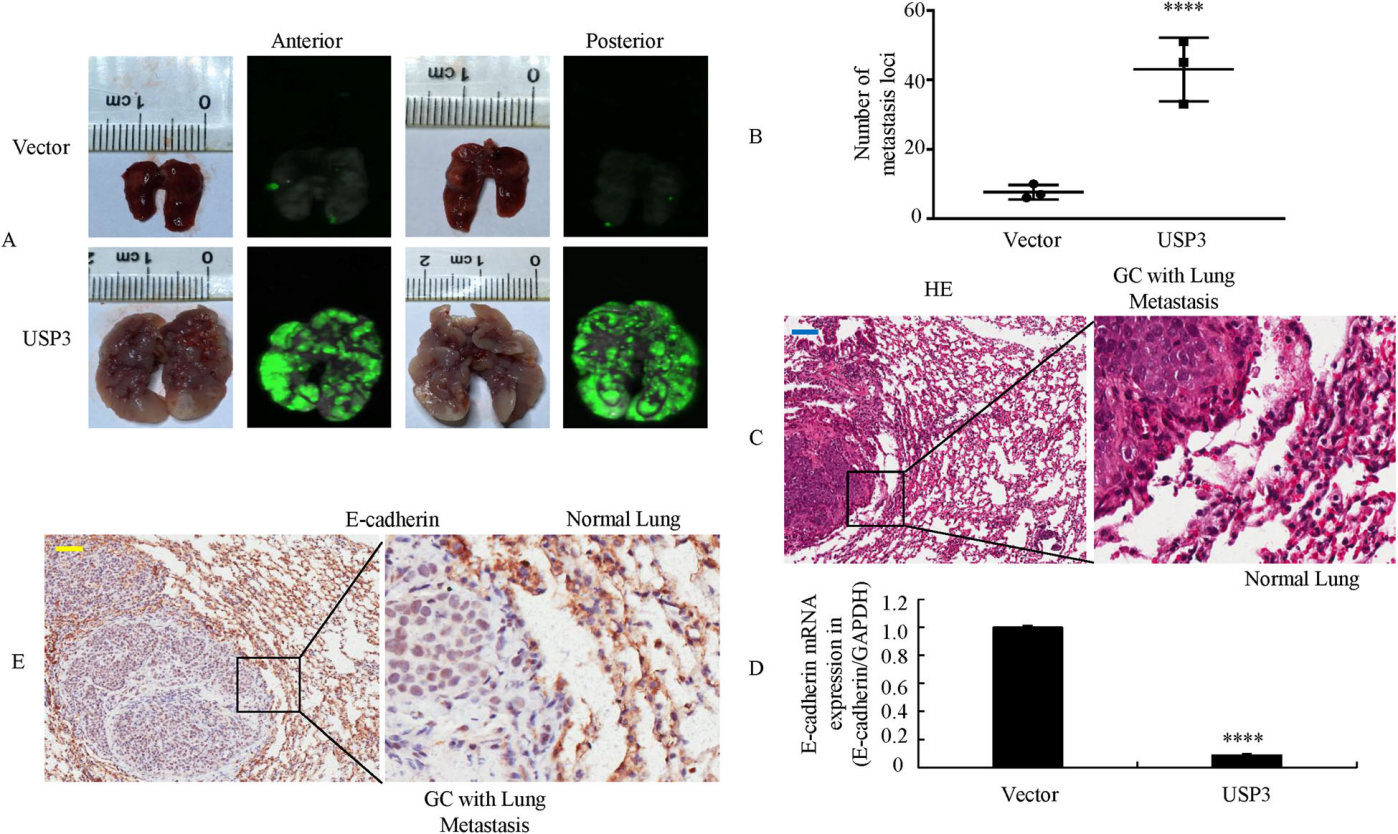
USP3 promotes EMT and metastasis in vivo. **a** External whole-body fluorescence images of mice orthotopically transplanted with BCG-823 cells (*n* = 3 in each group). Representative images of metastatic loci in the lungs are shown. **b** The number of metastatic loci in the lungs was counted. **c** Metastatic cancer tissues were stained with H&E. **d** Expression of E-cadherin positivity in tumors derived from BCG-823 cells was determined by qRT-PCR. ****, *P* < 0.001. **e** E-cadherin expression in the lung metastasis of GC was detected by IHC. Scale bars, 100 μm in **c** and **e**

To test the effects of USP3 on GC metastasis in vivo, the Lv-vector or Lv-USP3 cells were injected into the tail vein of nude mice. On day 34 after inoculation, the mice were sacrificed, the lungs were collected, and metastatic colonies were counted. The USP3-overexpressing cells formed a variety of large metastatic nodules in the lungs compared with the vector cells (Fig. 3a). The mean number of metastatic colonies in the USP3-overexpressing cells group indicated enhanced invasion compared with that in the vector cells (Fig. 3b). The presence of lung metastases from BGC-823 cells was confirmed by histological analysis (Fig. 3c).

We next tested whether USP3 affects the EMT process. Using qPCR, we found that USP3-overexpressing cells resulted in a substantial decrease in E-cadherin expression compared with vector cells (Fig. 3d). Additionally, the presence of GC metastases in the lungs was confirmed by IHC using anti-E-cadherin antibodies (Fig. 3e).

These findings suggest that ectopic USP3 expression is involved in the regulation of EMT and metastasis in GC cells.

### USP3 can influence the expression of SUZ12 protein in GC cells

**Fig. 4:**
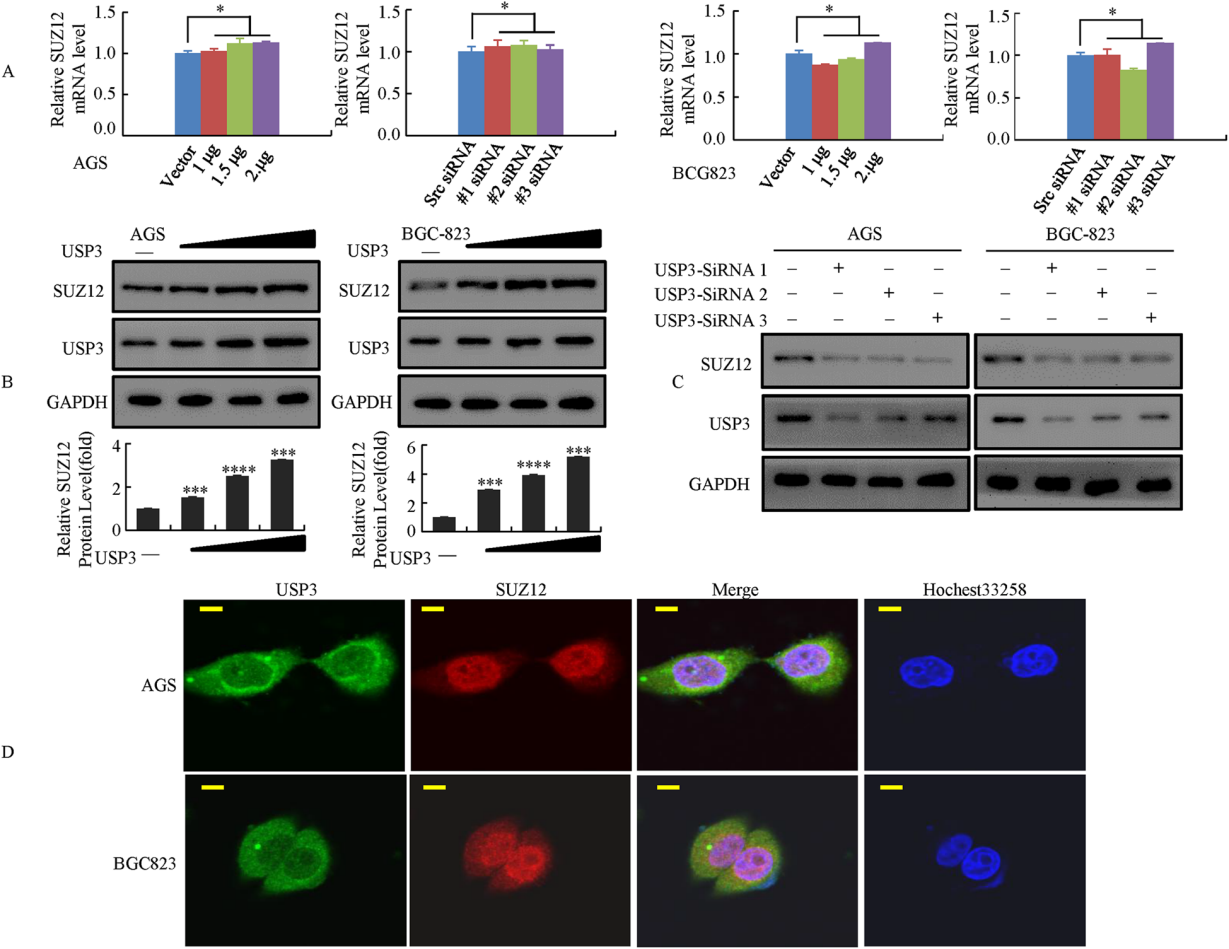
USP3 can influence the expression of SUZ12 protein in GC cells. **a** Overexpression of USP3 or the knock down of USP3 using three independent siRNAs did not affect the SUZ12 mRNA level in GC cells. *, *P* > 0.05. **b** Increasing amounts of USP3 were transfected into GC cells and SUZ12 expression was detected by Western blot. ***, *P* < 0.01; ****, P < 0.001. **c** The knock down of USP3 was achieved by transfecting cells with three independent siRNAs for 48 h. The expression of USP3 and SUZ12 was detected by Western blot. **d** Double staining for USP3 and SUZ12 in GC cells, as visualized by confocal microscopy, with the nuclei counterstained with Hoechst 33258. Scale bars represent 20 μm

To determine differentially expressed proteins that may serve as potential interaction targets of USP3, total protein extracted from the treated BGC-823 cells stably expressing the Flag-USP3 fusion protein and the BGC-823 cells carrying an empty vector was analyzed using iTRAQ experiments. In total, 5631 proteins (29,570 unique peptides) were confidently identified and were used for further quantification analysis. Using the criteria described in the “Material and methods”, 63 proteins were found to be downregulated (Additional file 1: Table S2) and 59 were found to be upregulated in the treated BGC-823 cells compared with the control cells (Table 1). Among them, SUZ12 was upregulated when the USP3 gene was ectopically expressed. Real-time PCR analysis suggested that overexpression of USP3 or the knock down of USP3 by siRNA had no effect on the SUZ12 mRNA level (Fig. 4a). However, according to Western blot analysis, endogenous SUZ12 was increased upon USP3 upregulation in GC cells (Fig. 4b) and reduced upon USP3 inhibition (Fig. 4c), which indicates that the regulatory effects of USP3 on SUZ12 expression only occur at the posttranscriptional level.

**Table 1:** iTRAQ ratios of up-regulated proteins in GC tissues

No.	Protein name	Gene name	Accession Number	Molecular Weight	Ratio
1	Muskelin	MKLN1	gi|31,881,798	85 kDa	2.23
2	Polypeptide N-acetylgalactosaminyltransferase 13	GLT13	gi|145,309,313	48 kDa	2.06
3	Keratin, type II cytoskeletal 1	K2C1	gi|375,314,775	66 kDa	2.01
4	Keratin, type I cytoskeletal 9	K1C9	gi|675,724,924	62 kDa	1.84
5	Ubiquitin carboxyl-terminal hydrolase 3	UBP3	gi|55,770,886	59 kDa	1.54
6	Histamine N-methyltransferase	HNMT	gi|5,901,970	33 kDa	1.53
7	Vomeronasal type-1 receptor 5	VN1R5	gi|27,777,675	41 kDa	1.5
8	Kinesin-associated protein 3	KIFAP3	gi|18,105,054	91 kDa	1.47
9	Zinc finger protein 592	ZN592	gi|108,860,697	137 kDa	1.47
10	Otogelin-like protein	OTOGL	gi|449,081,296	262 kDa	1.46
11	Period circadian protein homolog 2	PER2	gi|6,683,645	137 kDa	1.43
12	Collagen alpha-3(IX) chain	CO9A3	gi|426,392,464	63 kDa	1.39
13	RAS (RAD And GEM)-Like GTP-Binding 1	REM1	gi|724,948,224	33 kDa	1.39
14	Coiled-coil and C2 domain-containing protein 2A	C2D2A	gi|197,209,974	186 kDa	1.37
15	Kinesin-like protein KIF12	KIF12	gi|85,541,032	71 kDa	1.36
16	Prostate stem cell antigen	PSCA	gi|114,622,040	13 kDa	1.36
17	Conserved oligomeric Golgi complex subunit 2	COG2	gi|6,678,676	83 kDa	1.36
18	Adenomatous polyposis coli protein	APC	gi|53,759,122	312 kDa	1.35
19	Elongation of very long chain fatty acids protein 7	ELOV7	gi|157,388,947	145 kDa	1.34
20	SH3 domain-binding protein 4	SH3B4	gi|7,657,562	107 kDa	1.34
21	Keratin, type II cytoskeletal 5	K2C5	gi|119,395,754	62 kDa	1.31
22	Retinoblastoma-associated protein	RB	gi|108,773,787	106 kDa	1.31
23	Serine/threonine-protein kinase ULK3	ULK3	gi|656,214,624	31 kDa	1.3
24	Zinc finger protein 469	ZN469	gi|188,536,004	410 kDa	1.3
25	Kinetochore protein Spc25	SPC25	gi|10,190,716	26 kDa	1.3
26	Shugoshin 2	SGO2	gi|229,892,306	145 kDa	1.3
27	POTE ankyrin domain family member I	POTEI	gi|475,808,427	121 kDa	1.29
28	Myelin expression factor 2	MYEF2	gi|296,439,294	64 kDa	1.28
29	Mucolipin-3	MCLN3	gi|24,496,763	64 kDa	1.28
30	Putative heat shock protein HSP 90-beta 4	H90B4	gi|61,104,915	56 kDa	1.28
31	U6 snRNA-associated Sm-like protein LSm4	LSM4	gi|513,024,372	15 kDa	1.28
32	tRNA (adenine(58)-N(1))-methyltransferase, mitochondrial	TR61B	gi|222,831,587	53 kDa	1.27
33	Phosphatidate phosphatase LPIN1	LPIN1	gi|387,528,011	99 kDa	1.27
34	Uncharacterized protein C1orf167	CA167	gi|158,706,475	162 kDa	1.26
35	Nebulin	NEBU	gi|635,377,472	772 kDa	1.26
36	SPRY domain-containing protein 3	SPRY3	gi|14,249,554	31 kDa	1.26
37	Nuclear receptor 2C2-associated protein	NR2CA	gi|28,882,043	16 kDa	1.26
38	Charged multivesicular body protein 5	CHMP5	gi|189,409,150	25 kDa	1.25
39	Collagen alpha-5(VI) chain	CO6A5	gi|189,082,691	290 kDa	1.25
40	Dedicator of cytokinesis protein 11	DOC11	gi|145,699,123	215 kDa	1.25
41	BPI fold-containing family A member 3	BPIA3	gi|109,627,654	28 kDa	1.25
42	Obscurin	OBSCN	gi|403,501,446	925 kDa	1.24
43	CCR4-NOT transcription complex subunit 6	CNOT6	gi|28,872,863	63 kDa	1.24
44	5-methylcytosine rRNA methyltransferase NSUN4	NSUN4	gi|40,316,918	49 kDa	1.23
45	Gamma-interferon-inducible lysosomal thiol reductase	GILT	gi|6,165,618	28 kDa	1.23
46	POZ-, AT hook-, and zinc finger-containing protein 1	PATZ1	gi|586,477,589	70 kDa	1.23
47	Suppressor of zest 12	SUZ12	gi|197,333,809	83 kDa	1.23
48	MICOS Complex Subunit MIC27	MIC27	gi|116,812,610	29 kDa	1.22
49	SPARC-related modular calcium-binding protein 1	SMOC1	gi|11,545,873	48 kDa	1.22
50	Replication Termination Factor 2	RTF2	gi|193,788,722	34 kDa	1.22
51	Poly(A) polymerase alpha	PAPOA	gi|32,490,557	83 kDa	1.22
52	RNA-binding protein 42	RBM42	gi|545,830,327	50 kDa	1.22
53	Platelet-activating factor acetylhydrolase IB subunit beta	PAFAH1B2	gi|532,006,944	26 kDa	1.21
54	Ornithine decarboxylase antizyme 1	OAZ1	gi|576,067,926	25 kDa	1.21
55	Zinc finger and BTB domain-containing protein 11	ZBT11	gi|166,235,167	119 kDa	1.21
56	Methyltransferase-like protein 17, mitochondrial	MET17	gi|12,232,389	50 kDa	1.21
57	Glycogen synthase kinase-3 beta	GSK3B	gi|395,758,596	48 kDa	1.21
58	Thymosin beta-4	TYB4	gi|112,180,578	5 kDa	1.21
59	Chromosome transmission fidelity protein 18 homolog	CTF18	gi|27,501,458	129 kDa	1.21

Furthermore, the localization of USP3 and SUZ12 was visualized by confocal laser scanning microscopy. The results demonstrated that both proteins were localized to the same regions in GC cells. Overlapping fluorescence signals revealed that the two proteins were colocalized mainly in the nucleus and slightly in the cytoplasm (Fig. 4d).

Together, these findings indicate that USP3 should be a potent regulator of SUZ12 in GC cells.

### USP3 interacts with and deubiquitinates SUZ12

**Fig. 5:**
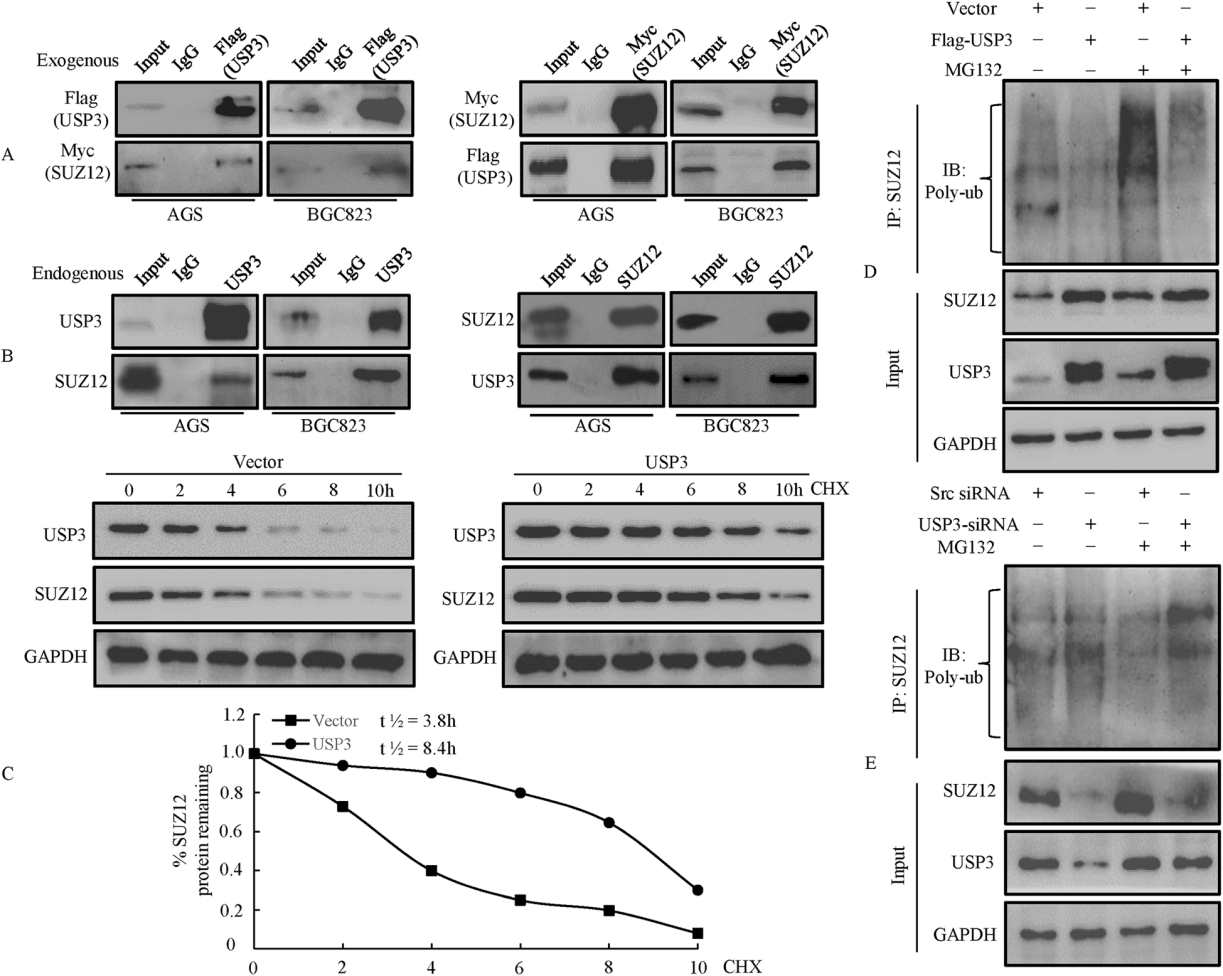
USP3 stabilizes SUZ12. **a** Flag-tagged USP3 plasmid was transfected into GC cells. Immunoprecipitation was performed with anti-flag antibody, and pre-immune normal mouse immunoglobulin G (nm IgG) was used as a control. Western blot analysis was performed with an anti-Myc (SUZ12) antibody. **b** Cell lysates of GC cells were immmunoprecipitated by an anti-USP3 antibody or the control antibody, normal rabbit immunoglobulin G (nr IgG). Western blotting was performed with an anti-SUZ12 antibody. All experiments were repeated 3 times, and similar findings were obtained. **c** Flag-USP3 was transfected into BCG-823 cells. After treating the cells with cycloheximide (CHX; 50 mg/mL) for the indicated time intervals, expression of SUZ12 and USP3 was examined by western blot (up) using the indicated antibodies. The intensity of endogenous SUZ12 expression for each time point was quantified by densitometry (down). **d** USP3 or vector was transfected into BCG-823 cells for 48 h. After the cells were treated with or without 10 mmol/L MG132 for 6 h, SUZ12 was immunoprecipitated with anti-SUZ12 antibody, and the polyubiquitination of SUZ12 was examined by Western blot using anti-ubiquitin. IP: immunoprecipitation; Wb: Western blot. **e** The scr or USP3 siRNA was transfected into BCG-823 cells for 48 h and then cells were treated with or without 10 mmol/L MG132 for 6 h. Extracts were immunoprecipitated with an anti-SUZ12 antibody, and the polyubiquitination of SUZ12 was examined by Western blot using anti-ubiquitin. The experiments were repeated three times, and representative images of the blots are shown

To elucidate the mechanisms responsible for the effects of USP3 on SUZ12, GC cells were cotransfected with Flag-tagged USP3 and Myc-tagged SUZ12 constructs. We found that Flag-tagged USP3 could be co-immunoprecipitated with Myc-tagged SUZ12 in GC cells. Similarly, Myc-tagged SUZ12 was co-immunoprecipitated using an anti-USP3 (Flag) antibody (Fig. 5a). To further confirm that USP3 was associated with the SUZ12 protein, we performed reciprocal co-immunoprecipitation experiments using endogenous protein and showed that USP3 can interact with SUZ12 (Fig. 5b). Collectively, these findings suggest that USP3 can physically interact with SUZ12.

To address whether and how USP3 stabilizes SUZ12, we treated vector and BCG-823 cells expressing USP3 with cycloheximide and prepared extracts after different time points (Fig. 5c). The SUZ12 protein level was decreased in vector cells with a half-life of approximately 3.8 h, which demonstrated that the protein was subjected to proteolysis. In contrast, the protein was essentially stable over the entire time period in cells expressing USP3 (estimated half-life of SUZ12: 8.4 h). These results suggested that USP3 enhanced SUZ12 stability by blocking protein degradation.

Since USP3 mediates the removal and processing of ubiquitin [26], it was speculated that USP3 directly regulates SUZ12 protein stability through deubiquitination of SUZ12. To assess this possibility, flag-tagged USP3 or empty vector plasmid was transfected into BCG-823 cells. Western blot showed that the ubiquitination of SUZ12 was strongly inhibited by USP3 expression in the presence or absence of MG132, a potent inhibitor of the 26S proteasome (Fig. 5d). Knockdown of USP3 expression led to opposite results (Fig. 5e).

Taken together, these findings suggest that USP3 is a DUB that controls the level of SUZ12 protein through interaction with and deubiquitination of SUZ12.

### SUZ12 is involved in USP3-induced GC cell migration, invasion and EMT

**Fig. 6:**
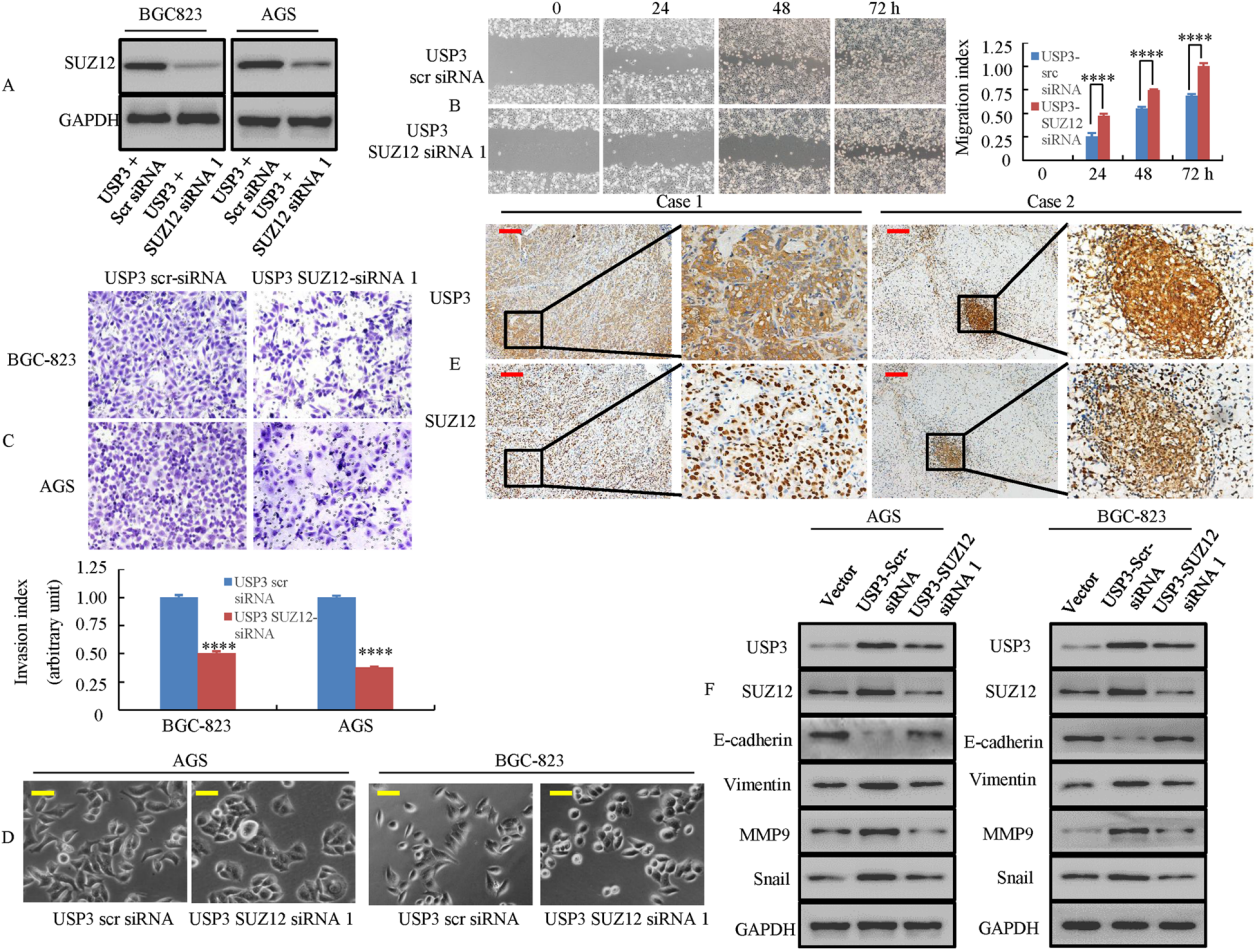
SUZ12 is involved in USP3-induced GC cell migration and invasion. **a** SUZ12 expression levels were detected using Western blot analysis in GC cells, which were transfected with USP3 overexpression plasmids. This was followed by transfection with SUZ12 siRNA1 or Scr siRNA. **b** Confluent AGS monolayers transfected with USP3 SUZ12 siRNA1 or USP3 scr siRNA were scratch- wounded at 72 h after transfection. Photographs were obtained 72 h after wound generation. ****, P < 0.001, USP3 scr siRNA vs. USP3 SUZ12 siRNA1. **c** Cell invasion of USP3 SUZ12 siRNA1 or USP3 Scr siRNA in GC cells was assessed by a Matrigel invasion chamber. The number of invaded cells in GC cells was counted. All values shown are mean ± SD of triplicate measurements and were repeated 3 times with similar results. ****, *P* < 0.001, USP3 Scr siRNA vs. USP3 SUZ12 siRNA1. **d** Morphology in USP3 SUZ12 siRNA1 or USP3 Scr siRNA cells as visualized by phase-contrast microscopy. **e** Representative IHC images are shown for USP3 and SUZ12 expression in lymph node metastatic cancer tissues. **f** Knock-down of SUZ12 in USP3-overexpressing cells inhibited the AKT/ERK/EMT signaling pathway as detected using Western blot analysis. Scale bars, 50 μm in **d** and 200 μm in **e**

To investigate the relationship among USP3, SUZ12 and the capacity for cell migration and invasion, the expression of SUZ12 in GC cells was downregulated by siRNA 1 (Fig. 6a). Intriguingly, the cell migratory and invasive abilities of GC cells transfected with SUZ12 siRNA 1 were significantly weakened (Fig. 6b & c).

Recently, it has been shown that upregulation of SUZ12 promotes metastasis through the regulation of EMT in cancer cells [16, 17]. We thus investigated whether SUZ12 affects the EMT process by regulating USP3 expression. We have indicated that USP3-transfected cells exhibited an elongated fibroblastoid phenotype by phase-contrast microscopy. However, SUZ12 knockdown in USP3 cells restored their normal phenotype, which consists of a round or flat morphology with short cytoplasmic processes (Fig. 6d). Furthermore, USP3 and SUZ12 expression was detected by IHC in metastatic tissues obtained from lymph nodes. We found that USP3 and SUZ12 were expressed at high levels in the nucleus and cytoplasm of cancer cells from two patients (Fig. 6e).

In addition, we performed a Western blot analysis and showed that knockdown of SUZ12 in USP3-overexpressing cells increased the expression of an epithelial marker (E-cadherin) but decreased the expression of mesenchymal markers (vimentin, Snail and MMP9) (Fig. 6f).

Taken together, these data suggest that the USP3-SUZ12 axis promotes an EMT-like phenotype in GC cells.

### Positive correlation between USP3 and SUZ12 expression in GC cells and GC tissues

**Fig. 7:**
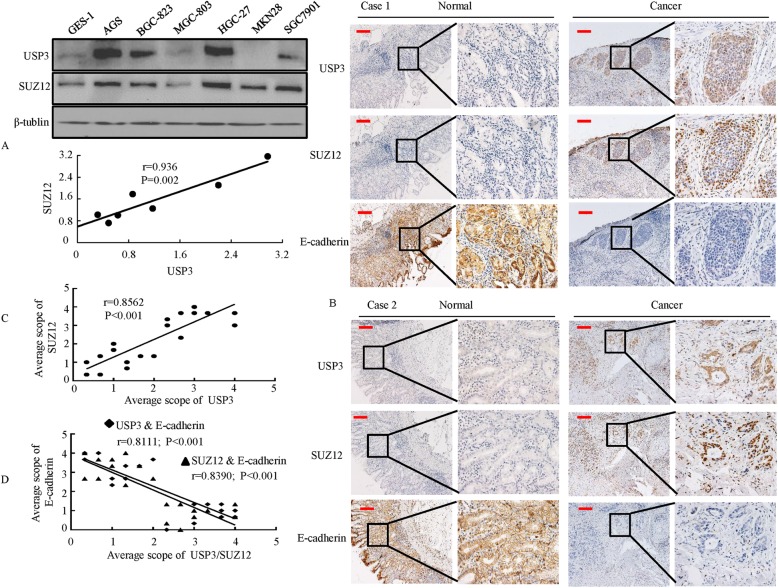
Expression of USP3, SUZ12 and E-cadherin proteins is correlated in GC cells and GC tissues. **a** Western blot analysis was performed to detect the expression of USP3 and SUZ12 in GC cells. Spearman’s correlation analysis between USP3 and SUZ12 protein levels in GC cells. **b** USP3, SUZ12 and E-cadherin expression levels in ten normal and cancerous gastric tissue specimens were detected by IHC. **c** & (**d**) Spearman’s correlation analysis was used to determine the relationship between SUZ12 and USP3 (**c**), that between SUZ12 and E-cadherin (**d**), and that between USP3 and E-cadherin (**d**) protein expression in normal and cancerous gastric tissue specimens. Scale bars, 50 μm in B

To further examine the relationship between USP3 and SUZ12, the expression of USP3 and SUZ12 was analyzed in GC cells and tissues. The results showed that the protein levels of USP3 and SUZ12 were positively correlated in the majority of these GC cells (Fig. 7a). Immunohistochemical staining showed that USP3- and SUZ12-positive signals were strongly or moderately expressed in the carcinoma cells of all ten GC samples as exemplified in Fig. 7b. Moreover, the expression profiles of USP3 and SUZ12 in cancerous and normal gastric tissues were inversely correlated with the profile of the epithelial marker E-cadherin (Fig. 7b). USP3 and SUZ12 were only expressed in cancer cells, whereas E-cadherin was predominantly expressed in normal epithelial cells (Fig. 7b). In addition, Spearman’s rank correlation analysis confirmed that USP3 protein expression was positively associated with SUZ12 protein expression in human tissue (Fig. 7c). The levels of USP3 and SUZ12 proteins were negatively correlated with the levels of E-cadherin protein (Fig. 7d).

Together, these results demonstrate a positive correlation between USP3 and SUZ12 expression levels and a negative correlation between the levels of USP3/SUZ12 and E-cadherin protein.

## Discussion

In this study, we report on USP3, a gene that is overexpressed in various cancers, including GC and that acts as an important oncogene. USP3 increased cell migration, invasion and the ectopic expression of USP3 in GC cells and induced EMT in vitro and in vivo. Moreover, USP3 interacts with, depolyubiquitylates and stabilizes SUZ12. Co-expression of USP3 and SUZ12 induces cell metastasis through the induction of EMT. These findings demonstrate that USP3 is an essential regulator of SUZ12 and that the USP3-SUZ12 signaling axis plays a critical role in the progression and metastasis of GC.

USP3 is a member of the ubiquitin-specific protease (USP) subfamily of deubiquitinating enzymes (DUBs) [8–10]. Previous studies have revealed its potential roles in the molecular pathophysiology of cancer. Here, we provided evidence that the GC tissues had higher USP3 expression relative to adjacent adjacent nontumor tissues, which further confirmed the findings of previous studies. A significant relationship was observed between elevated USP3 expression and differentiation, AJCC stage and lymph node metastasis. Moreover, high expression of USP3 predicted poor survival in GC patients according to a survival analysis. In addition, USP3 overexpression promoted GC cell migration and invasiveness. These findings indicated a potential relationship between USP3 expression and an aggressive phenotype in GC.

EMT is implicated in the initial step of cancer metastasis and is indispensable for cancer cells to break through the barriers formed by normal tissues so that a tumor can metastasize [20, 23, 27]. The heterogeneity in GC also increases the risk of gastric carcinoma progression and metastasis and exacerbates therapeutic resistance [28]. Recently, of the proteins in the ubiquitin-specific protease (USP) family, USP10 was found to induce EMT and aggressiveness in human cancer [29, 30]. Consistently, we have shown that USP3 overexpression caused the loss of epithelial polarity and the expression of EMT markers including the following: loss of the epithelial cell marker E-cadherin and upregulation of the mesenchymal cell marker vimentin. Moreover, ectopic expression of USP3 led to the acquisition of molecular and functional characteristics of EMT, which caused the cells to appear scattered. This phenomenon is consistent with the previous theory that EMT is essential for the dissemination of tumor cells from adjacent tissues and for the seeding of new tumors at distant sites [31]. Hence, our study provides significant insights into the novel roles of USP3 as a factor that promotes GC metastasis through EMT.

TGF-β1 is a multifunctional cytokine that regulates a wide range of cellular functions. TGF-β1-stimulates cells to become spindle-shaped and undergo morphological changes such as a decrease in cell-cell adhesion and loss of polarity [24, 25]. TGF-β1 is thought to be a key factor that contributes to cancer progression, primarily via EMT-triggered metastasis [25, 32]. Studies have reported that some members of the USP gene family have a role in mediating TGF-β-induced EMT. Kit Leng Lui S et al. showed that USP26 regulates TGF-β signaling by deubiquitinating and stabilizing SMAD7 [33]. Moreover, TGF-β upregulates the translation of USP15 via the PI3K/AKT pathway to promote p53 stability [34]. Among the USP genes, we found that induction of EMT in GC cells by TGF-β1 was associated with a significant increase in USP3 expression. When USP3 expression was silenced, the EMT phenotype induced by TGF-β1 was abrogated, and USP3 expression was also decreased. This is the first study that has explored the effect of USP3 on TGF-β1-induced EMT in GC.

To better understand the effect of USP3 on quantitative protein changes in GC, we utilized differential proteomics, which consisted of Isobaric Tag for Relative and Absolute Quantitation (iTRAQ) in combination with LC-MS/MS. iTRAQ has gained popularity for its ability to perform concurrent identification and relative quantification of hundreds of proteins; iTRAQ has also been employed in several oncoproteomics studies [35–37]. In the current study, iTRAQ data showed that the differentially expressed proteins (59 upregulated and 63 downregulated) were widely distributed in the cell and demonstrated that they played a role in all aspects of cell biology, which indicates that USP3 is involved in almost all biological processes.

As mentioned above, SUZ12 was chosen for further study. SUZ12, a core component of the Polycomb PRC2-HMTase complex, plays an oncogenic role in GC [11, 12]. Previous studies have indicated that overexpression of SUZ12 is associated with migration and invasion through its regulation of EMT in tumors [16, 17]. Recently, researchers have shown that SUZ12 was transcriptionally regulated by some proteins, including NF-κB and E2F1 [38]. However, the mechanism by which SUZ12 protein stability and turnover are regulated remains unknown. In the current study, SUZ12 was identified as one of USP3-interacting proteins, which was confirmed by co-IP and confocal laser scanning microscopy analysis.

Sharma N et al. have found that USP3 counteracts RNF168 via deubiquitinating H2A and γH2AX at lysines 13 and 15 [39]. Fu S and his colleagues have shown that USP3 interacts with p53 and regulates p53 stability and that depletion of USP3 leads to accelerated degradation of p53 in normal cells and therefore enhanced cell proliferation and transformation [40]. Consistently, overexpression of USP3 stabilizes the SUZ12 protein by binding to and deubiquitinating SUZ12 and subsequently promoting GC progression. These findings are consistent with the hypothesis that regulation of SUZ12 by USP3 is a posttranslational event.

To determine the relationship between expression of USP3 and SUZ12, SUZ12 was downregulated in USP3-overexpressing cells using siRNA. Intriguingly, the knock down of SUZ12 reduced the capability of cell migration and invasion compared with the USP3-expressing cells, which suggests that USP3 exerted its effects at least in part by deubiquitinating and stabilizing SUZ12. Moreover, SUZ12 knockdown in USP3-expressing cells restored their normal morphology and inhibited the expression of vimentin, Snail and MMP-2/9. Thus, the results suggest that the induction of EMT by SUZ12 is mediated by USP3. In addition, we observed that the distribution pattern of USP3 was highly congruent with that of SUZ12 in GC cells and clinical GC samples. For this reason, it is hypothesized that USP3 drives GC invasion, metastasis and EMT through a mechanism wherein USP3 stabilizes SUZ12 protein.

## Conclusion

In conclusion, our study shows that USP3 could promote cell migration, invasion and EMT in GC cells through interacting with and deubiquitinating SUZ12. Thus, targeting the USP3-SUZ12 axis may provide a potential therapeutic target for the treatment of GC.

## Additional files

Additional file 1: **Table S1.** Correlation between USP3 protein expression and the clinicopathological parameters of GC. **Table S2.** iTRAQ ratios of down-regulated proteins in GC tissues. **Supplementary materials and methods.** (DOCX 53 kb)

Additional file 2: **Figure S1.** Functional analysis of USP3 in vitro. **(A)** Invasive potential of the GC cells transfected with the USP3 or Vector. **, *P* < 0.05; ****, *P* < 0.001. **(B) (C) & (E)** For the wound-healing experiments, cells were analyzed with live-cell microscopy. ****, *P* < 0.001**. (D)** Invasive potential of the GC cells transfected with USP3 siRNA or src siRNA. ****, *P* < 0.001**. (F)** USP3 is regulated by the AKT/ERK/EMT signaling pathway in GC cells according to the Western blot analysis. (TIF 808 kb)

Additional file 3: **Figure S2.** Overexpression of USP3 enhances TGF-β1-induced EMT. **(A and B)** BCG-823 cells were pretreated with various concentrations of TGF-β1 (0–2 ng/ml) for 48 h (A) or with TGF-β1 (1 ng/ml) for 48 h (B) and then subjected to western blot to detect E-cadherin, vimentin and USP3. GAPDH was used as the internal control. **(C)** BCG-823 cells were treated with recombinant TGF-β1 (2 ng/ml) in the presence of neutralizing anti-TGF-β1 antibody (a-TGF-β, 2 mg/ml) or mouse IgG (mIgG) for 48 h. **(D)** Cells in which USP3 was knocked down or transfected with scr siRNA were treated with g-TGF-β1 for an additional 48 h. The expression of USP3, E-cadherin and vimentin was detected by Western blot. **(E)** The morphology of GC cells was observed under an inverted microscope. (**F**) The GC cells were transfected with USP3 siRNA or scr siRNA for 24 h followed by TGF-β1 treatment for 24 h. Representative images and data from a Transwell assay in GC cells. Each bar represents the mean ± SD. ****, *P* < 0.001, compared with GC cells treated with TGF-β1. ****, *P* < 0.001, compared with cells transfected with USP3 siRNA and treated with TGF-β1. The error bars represent the mean ± SD from 3 independent experiments. Scale bars, 50 μm in E. (TIF 4576 kb)

## Data Availability

All remaining data are available within the article and supplementary files, or available from the authors upon request.

## Abbreviations

ATCC: American Type Culture Collection
DUBs: deubiquitinating enzymes
EMT: epithelial-to-mesenchymal transition
IHC: immunohistochemistry
iTRAQ: Isobaric Tag for Relative and Absolute Quantitation
siRNA: short interfering RNA
SUZ12: suppressor of zeste 12 protein homolog
USP3: ubiquitin-specific protease 3
