# Supplementary material for: Ubiquitin-specific protease 3 promotes cell migration and invasion by interacting with and deubiquitinating SUZ12 in gastric cancer
Source: J Exp Clin Cancer Res. 2019 Jun 24;38:277. doi: 10.1186/s13046-019-1270-4 (PMC6591922; doi:10.1186/s13046-019-1270-4)
Supplement: Supplementary file 1 — Table S1. Correlation between USP3 protein expression and the clinicopathological parameters of GC. Table S2. iTRAQ ratios of down-regulated proteins in GC tissues. Supplementary materials and methods. (DOCX 53 kb) [file 13046_2019_1270_MOESM1_ESM.docx]

Additional file 1

Table S1. Correlation between USP3 protein expression and the clinicopathological parameters of GC

| Features | Total number (n =87 ) | USP3 Expression | | *P* |
| --- | --- | --- | --- | --- |
|  |  | Low | High |  |
| Age (years) |  |  |  | 0.383 |
| <55 | 17 | 7 | 10 |  |
| ≥55 | 70 | 20 | 50 |  |
| Gender |  |  |  | 0.808 |
| Male | 59 | 19 | 40 |  |
| Female | 28 | 8 | 20 |  |
| Differentiation |  |  |  | 0.000 |
| Well | 15 | 11 | 4 |  |
| Moderate | 45 | 15 | 30 |  |
| Poor | 27 | 1 | 26 |  |
| Lymph node metastasis |  |  |  | 0.013 |
| Yes | 67 | 16 | 51 |  |
| No | 20 | 11 | 9 |  |
| Tumor size (cm) |  |  |  | 0.016 |
| <10 | 16 | 11 | 5 |  |
| ≥10 | 71 | 16 | 55 |  |
| AJCC T stage |  |  |  | 0.029 |
| T1, T2 | 13 | 8 | 6 |  |
| T3, T4 | 74 | 19 | 54 |  |
| Clinical TNM stage |  |  |  | <0.001 |
| I，II | 31 | 16 | 15 |  |
| III，IV | 56 | 11 | 45 |  |

Table S2. iTRAQ ratios of down-regulated proteins in GC tissues

| No. | Protein name | Gene name | Accession  Number | Molecular  Weight | Ratio |
| --- | --- | --- | --- | --- | --- |
| 1 | Hemoglobin subunit beta | HBB | gi\|4504349 | 16 kDa | 0.45 |
| 2 | Maestro heat-like repeat-containing protein family member 2B | MRO2B | gi\|154240671 | 180 kDa | 0.51 |
| 3 | Heparan sulfate glucosamine 3-O-sulfotransferase 6 | HS3S6 | gi\|685504951 | 37 kDa | 0.58 |
| 4 | CBP80/20-dependent translation initiation factor | CTIF | gi\|40788256 | 68 kDa | 0.6 |
| 5 | Valine--tRNA ligase, mitochondrial | SYVM | gi\|268370297 | 118 kDa | 0.64 |
| 6 | Solute carrier family 15 member 4 | S15A4 | gi\|21717816 | 62 kDa | 0.67 |
| 7 | DNA endonuclease RBBP8 | CTIP | gi\|4506441 | 102 kDa | 0.67 |
| 8 | Leucine-rich colipase-like protein 1 | LRCL1 | gi\|306922388 | 18 kDa | 0.69 |
| 9 | Protein SCAI | SCAI | gi\|221554543 | 70 kDa | 0.69 |
| 10 | GRB2-associated and regulator of MAPK protein 2 | GARE2 | gi\|37700107 | 93 kDa | 0.72 |
| 11 | Protein MAL2 | MAL2 | gi\|16418397 | 19 kDa | 0.73 |
| 12 | Myosin-binding protein H-like | MBPHL | gi\|194018539 | 39 kDa | 0.73 |
| 13 | Coordinator of PRMT5 and differentiation stimulator | COPRS | gi\|217416381 | 20 kDa | 0.73 |
| 14 | Zinc finger protein 787 | ZN787 | gi\|694977208 | 40 kDa | 0.73 |
| 15 | High affinity copper uptake protein 1 | COPT1 | gi\|197102418 | 21 kDa | 0.73 |
| 16 | Zinc finger protein 670 | ZN670 | gi\|15082252 | 44 kDa | 0.74 |
| 17 | Scavenger receptor class A member 3 | SCAR3 | gi\|6230372 | 65 kDa | 0.74 |
| 18 | Prenylated Rab acceptor protein 1 | PRAF1 | gi\|222144309 | 21 kDa | 0.75 |
| 19 | DNA-directed RNA polymerases I, II, and III subunit RPABC2 | RPAB2 | gi\|591305623 | 14 kDa | 0.75 |
| 20 | tRNA (guanine-N(7)-)-methyltransferase | TRMB | gi\|167004078 | 31 kDa | 0.76 |
| 21 | Protein unc-13 homolog C | UN13C | gi\|122937514 | 251 kDa | 0.77 |
| 22 | Serine/threonine-protein phosphatase 2B catalytic subunit gamma isoform | PP2BC | gi\|21361290 | 58 kDa | 0.78 |
| 23 | Acetyl-coenzyme A transporter 1 | ACATN | gi\|4757708 | 61 kDa | 0.78 |
| 24 | MFS-type transporter SLC18B1 | S18B1 | gi\|16445357 | 49 kDa | 0.78 |
| 25 | Choline/ethanolamine kinase | CHKB | gi\|6978649 | 45 kDa | 0.78 |
| 26 | OCIA domain-containing protein 2 | OCAD2 | gi\|62244044 | 17 kDa | 0.78 |
| 27 | Laminin subunit alpha-2 | LAMA2 | gi\|215274259 | 344 kDa | 0.78 |
| 28 | High affinity cGMP-specific 3',5'-cyclic phosphodiesterase 9A | PDE9A | gi\|4505675 | 68 kDa | 0.79 |
| 29 | Origin recognition complex subunit 3 | ORC3 | gi\|32483367 | 82 kDa | 0.79 |
| 30 | Plexin-A4 | PLXA4 | gi\|157738645 | 212 kDa | 0.8 |
| 31 | Protein bassoon | BSN | gi\|91208420 | 416 kDa | 0.8 |
| 32 | KAT8 regulatory NSL complex subunit 2 | KANL2 | gi\|635064981 | 55 kDa | 0.8 |
| 33 | Methylcytosine dioxygenase TET2 | TET2 | gi\|187761317 | 223 kDa | 0.8 |
| 34 | Uncharacterized protein KIAA1671 | K1671 | gi\|223633988 | 197 kDa | 0.8 |
| 35 | ETS-related transcription factor Elf-4 | ELF4 | gi\|4503555 | 71 kDa | 0.8 |
| 36 | Nuclear cap-binding protein subunit 2 | NCBP2 | gi\|635086456 | 18 kDa | 0.8 |
| 37 | NACHT domain- and WD repeat-containing protein 1 | NWD1 | gi\|313104204 | 175 kDa | 0.81 |
| 38 | Tumor suppressor candidate 3 | TUSC3 | gi\|617563720 | 40 kDa | 0.81 |
| 39 | G patch domain-containing protein 8 | GPTC8 | gi\|50962882 | 164 kDa | 0.81 |
| 40 | Fibroblast growth factor 9 | FGF9 | gi\|344284636 | 23 kDa | 0.81 |
| 41 | Pleckstrin homology-like domain family A member 2 | PHLA2 | gi\|109105025 | 17 kDa | 0.81 |
| 42 | ATP synthase subunit a | ATP6 | gi\|700278955 | 25 kDa | 0.81 |
| 43 | Guanine nucleotide-binding protein G(olf) subunit alpha | GNAL | gi\|215272301 | 44 kDa | 0.81 |
| 44 | Exonuclease 3'-5' domain-containing protein 2 | EXD2 | gi\|301129155 | 70 kDa | 0.81 |
| 45 | Histone-lysine N-methyltransferase EHMT1 | EHMT1 | gi\|530426793 | 141 kDa | 0.81 |
| 46 | Chromodomain-helicase-DNA-binding protein 5 | CHD5 | gi\|24308089 | 223 kDa | 0.82 |
| 47 | Putative Polycomb group protein ASXL2 | ASXL2 | gi\|37998953 | 154 kDa | 0.82 |
| 48 | Keratin, type II cuticular Hb6 | KRT86 | gi\|530400236 | 54 kDa | 0.82 |
| 49 | Ubiquitin thioesterase OTUB2 | OTUB2 | gi\|12962939 | 27 kDa | 0.82 |
| 50 | Hepatocyte growth factor receptor | MET | gi\|42741655 | 156 kDa | 0.82 |
| 51 | Ubiquitin-conjugating enzyme E2 variant 3 | UEVLD | gi\|103472011 | 19 kDa | 0.82 |
| 52 | Ras-related protein Rab-22A | RB22A | gi\|10190714 | 22 kDa | 0.82 |
| 53 | UDP-glucuronosyltransferase 1-10 | UD110 | gi\|29789078 | 60 kDa | 0.82 |
| 54 | Transmembrane protein 223 | TM223 | gi\|18088875 | 22 kDa | 0.82 |
| 55 | Actin-related protein 2/3 complex subunit 3 | ARPC3 | gi\|77735497 | 21 kDa | 0.82 |
| 56 | Putative glycerol kinase 5 | GLPK5 | gi\|88196792 | 59 kDa | 0.83 |
| 57 | Four and a half LIM domains protein 3 | FHL3 | gi\|54112385 | 31 kDa | 0.83 |
| 58 | Chloride intracellular channel protein 2 | CLIC2 | gi\|169404567 | 28 kDa | 0.83 |
| 59 | tRNA-dihydrouridine(16/17) synthase [NAD(P)(+)]-like | DUS1L | gi\|40807366 | 53 kDa | 0.83 |
| 60 | Phosphomannomutase 1 | PMM1 | gi\|194097356 | 30 kDa | 0.83 |
| 61 | Synaptosomal-associated protein 23 | SNP23 | gi\|18765729 | 23 kDa | 0.83 |
| 62 | Microspherule protein 1 | MCRS1 | gi\|29893564 | 52 kDa | 0.83 |
| 63 | Serine/threonine-protein phosphatase PP1-gamma catalytic subunit | PP1G | gi\|4506007 | 37 kDa | 0.83 |

**Supplementary materials and methods.**

**Cells culture, antibodies and reagents.**

Human gastric carcinoma cell line AGS (ATCC, Rockville, MD) and BGC-823 (Beijing Institute of Cancer Research, Beijing, China) were maintained in DMEM basic containing 10% fetal calf serum (FCS, Invitrogen, Carlbad) at 37°C and 5% CO_2_.

Mouse anti-human FLAG (F3165) was product of Sigma (St Louis, MO). Mouse anti-human myc (Ab-1) was product of Calbiochem Corp (La Jolla, CA). Rabbit anti-USP3 (12490-1-AP), SUZ12 (51064-2-AP), UBC(Ubiquitin) (10201-1-AP), Ki-67 (27309-1-AP), E-cadherin (20874-1-AP), MMP9 (1037501-AP) and mouse anti-Vimentin (60330-1-lg) were purchased from Proteintech (Chicago, USA). Rabbit anti-GAPDH was purchased from Abcam (Abcam, Cambridge, UK). Rabbit anti-Akt (4685S), p-Akt (4060S), Erk1/2 (4695S), p-Erk1/2 (4370S) and Flag (14793S) were purchased from Cell Signaling (Massachusetts, USA). Recombinant human TGF-β1 (240-B) and monoclonal anti-TGF-β1 antibody were purchased from R&D Systems (Minneapolis, MN).

**Co-immunoprecipitations and Western blotting analysis**

The GC cells were lysed in NP-40 buffer and then were centrifuged for 20 min. To detect the polyubiquitylation of SUZ12, cells were lysed in RIPA buffer [50 mM tris-HCl (pH 7.4), 150 mM NaCl, 1% (v/v) NP-40, 0.5% deoxycholate, 0.1% SDS, and protease inhibitor cocktail]. For co-immunoprecipitations, 1 ml of postcentrifuged lysates was incubated with 0.5 to 2.5 *μ*g of antibody at 4℃ overnight, followed by incubation with a 50% slurry of protein A/G agarose (Amersham) for 2 hours at 4℃. Immunoprecipitated proteins were extensively washed with lysis buffer and were eluted with SDS-PAGE loading buffer by boiling for 5 min. For Western blotting analysis, proteins were resolved by SDS-PAGE and were transferred onto polyvinylidene difluoride membranes. Primary antibody (anti-FLAG, anti-Myc, anti-HA, anti-USP3 and anti- SUZ12) incubations were carried out overnight at 4˚C, followed by their respective secondary antibodies. GAPDH was used as a loading control. The membranes were washed 3 times with TBST and visualized with chemiluminescence (Beyotime, Beijing, China).

**Quantitative RT-qPCR.**

Total RNA was isolated from the cells and tissues using TRIzol reagent, according to the manufacturer's instructions (Invitrogen). cDNA was generated from 1 mg total RNA using SuperScript III Invitrogen) and polyN primers. qPCR was performed using the StepOne and StepOne Plus real-time PCR system (Applied Biosystems, Foster City, CA, USA). Glyceraldehyde 3-phosphate dehydrogenase (GAPDH) was used as a loading control. The primer sequences were as follows: E-cadherin forward, 5'-GCCCAGAAAATGAAAAAGG-3' and reverse, 5'- GTGTATGTGGCAATGCGTTC -3'; GAPDH forward, 5'- GTCAACGGATTTGGTCGTATTG -3' and reverse, 5'- CTCCTGGAAGATGGTGATGGG 3'. The relative levels of gene expression were represented as 2-^Δ^Ct (Ct gene - Ct reference). The experiments were repeated in triplicate.

**Wound-healing assays**

The cells were seeded into 24-well plates containing coverslips. The confluent monolayers were wounded in a line across the slides with a sterile 20 μL plastic pipette tips. All cellular debris was removed by washing the coverslips with

phosphate buffer saline (PBS). The distances migrated by the cell monolayer to close the wounded area during the indicated time were measured. Cell migration, indicating wound-healing effect, is here expressed as a migration index, i.e., the distance

migrated by GC cell at the indicated point in time relative to the initial length of the wound. Experiments were performed in at least triplicate.

**Invasion assays**

In brief, 200 μL of cell suspension (1 X 10^5^ cells) was added to the upper compartment of migration chambers (BD Biosciences). The bottom chamber was filled with 500 μL DMEM medium with 10% FBS. Then, 48 hours later, cells were fixed with 100% methanol and stained with 2% crystal violet. The number of invading cells was counted in five representative fields (200×) per insert.

**Immunofluorescence staining and confocal laser scanning microscopy**

When they were 60% confluent, the cells were plated onto sterilized glass coverslips, washed in phosphate-buffered saline (PBS) and fixed in acetone/carbinol (V/V=1/1) on ice for 2 min. The slides were then blocked in PBS plus 5% fetal bovine serum (FBS) at 37 °C for 1 h, followed by an incubation with anti-USP3 (Flag) and anti-SUZ12 (Myc) antibodies at 4 °C overnight. Next, the slides were washed three times with PBS-T (PBS plus 0.2% Triton X-100). For double immunofluorescence staining, the slides were incubated with rhodamine-conjugated anti-mouse IgG and fluorescein isothiocyanate (FITC)-conjugated anti-rabbit IgG at 37 °C for 1 h. The slides were then stained briefly with DAPI, washed with PBS-T, mounted, and analyzed via a confocal laser scanning microscope (Oberkochen, Germany).
