# Supplementary figures and images for: Ubiquitin-specific protease 3 promotes cell migration and invasion by interacting with and deubiquitinating SUZ12 in gastric cancer
Source: J Exp Clin Cancer Res. 2019 Jun 24;38:277. doi: 10.1186/s13046-019-1270-4 (PMC6591922; doi:10.1186/s13046-019-1270-4)

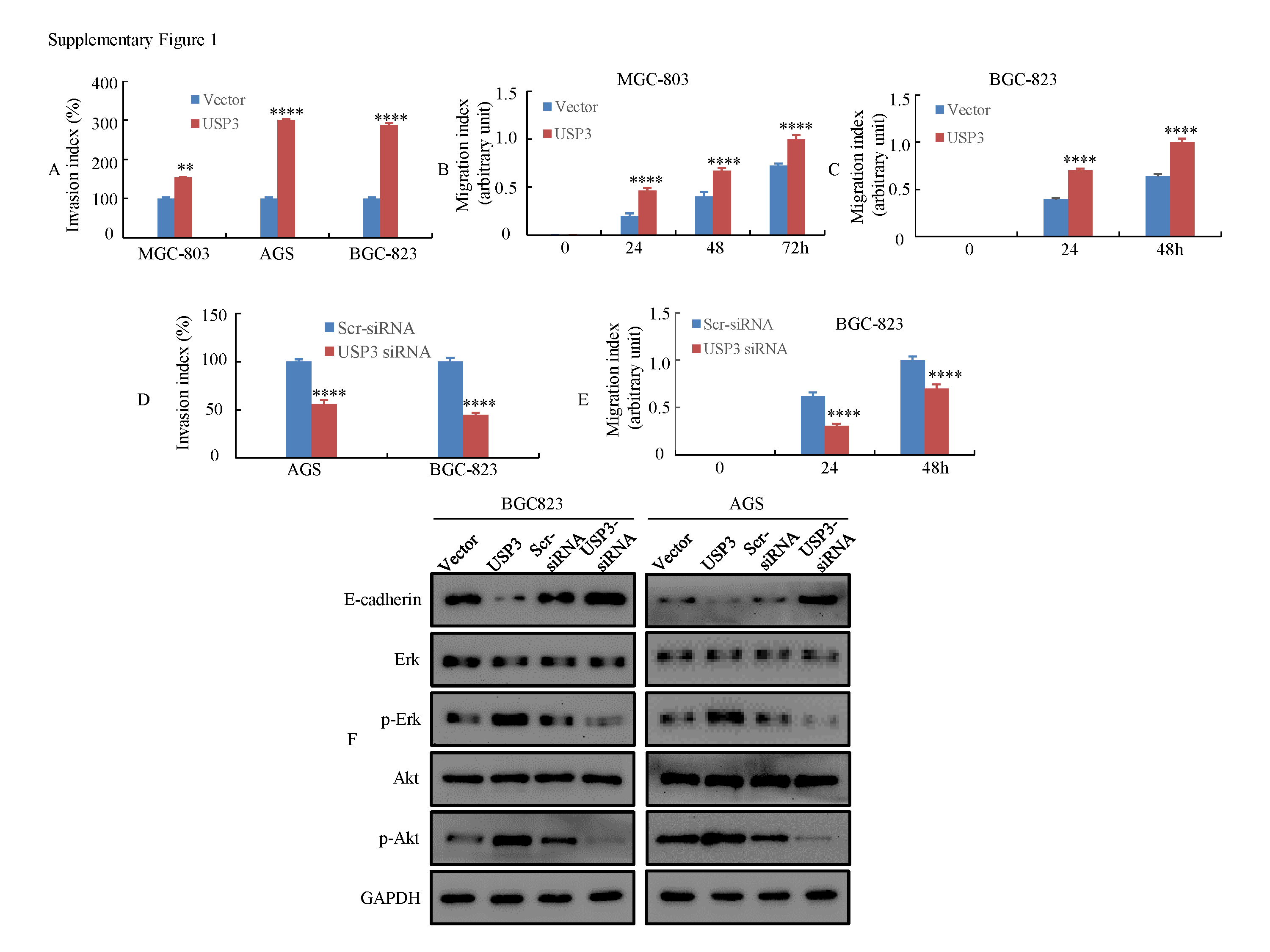

Supplement: Supplementary file 2 — Figure S1. Functional analysis of USP3 in vitro. (A) Invasive potential of the GC cells transfected with the USP3 or Vector. **, P < 0.05; ****, P < 0.001. (B) (C) & (E) For the wound-healing experiments, cells were analyzed with live-cell microscopy. ****, P < 0.001. (D) Invasive potential of the GC cells transfected with USP3 siRNA or src siRNA. ****, P < 0.001. (F) USP3 is regulated by the AKT/ERK/EMT signaling pathway in GC cells according to the Western blot analysis. (TIF 808 kb) [file 13046_2019_1270_MOESM2_ESM.tif]

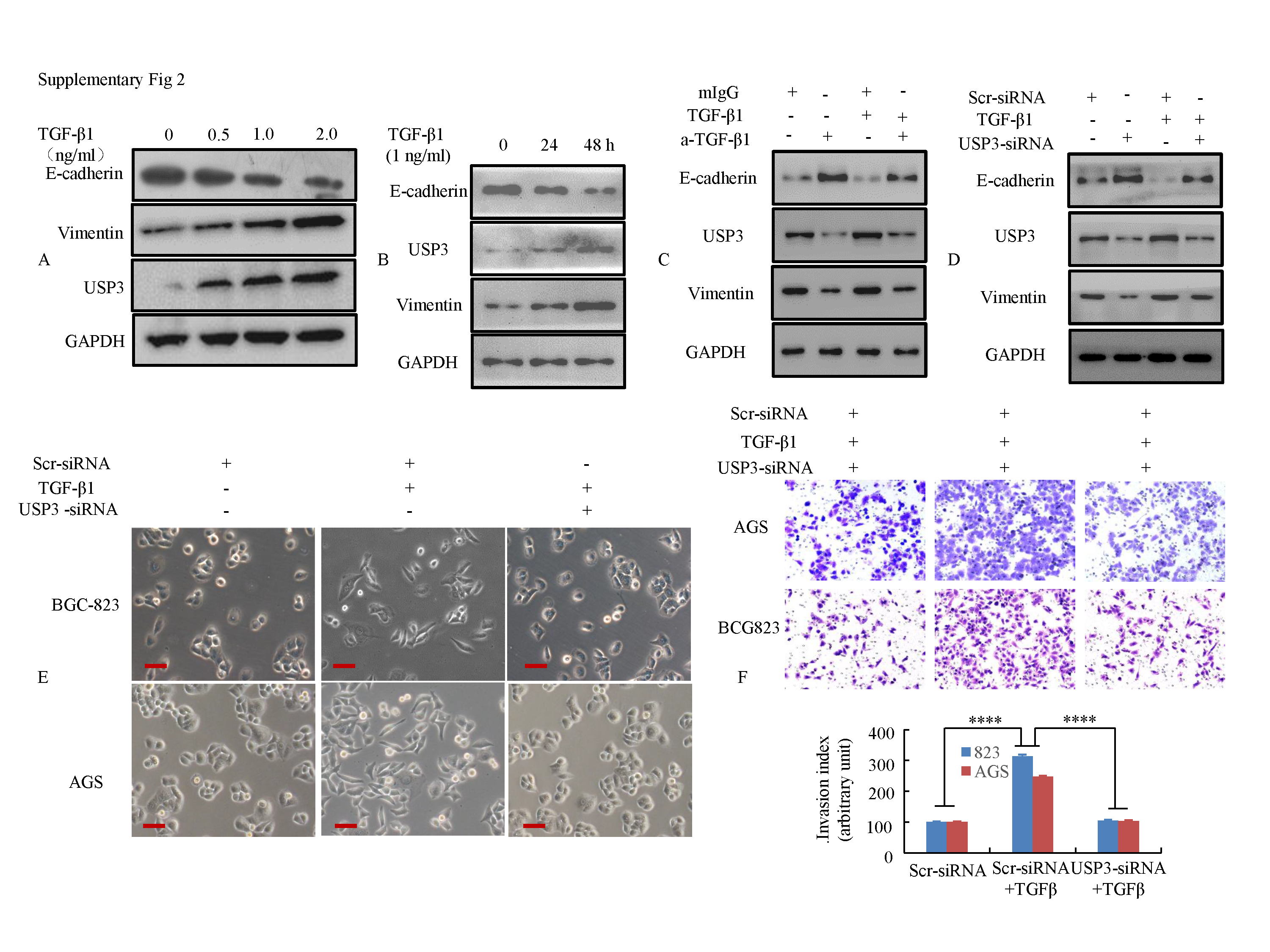

Supplement: Supplementary file 3 — Figure S2. Overexpression of USP3 enhances TGF-β1-induced EMT. (A and B) BCG-823 cells were pretreated with various concentrations of TGF-β1 (0–2 ng/ml) for 48 h (A) or with TGF-β1 (1 ng/ml) for 48 h (B) and then subjected to western blot to detect E-cadherin, vimentin and USP3. GAPDH was used as the internal control. (C) BCG-823 cells were treated with recombinant TGF-β1 (2 ng/ml) in the presence of neutralizing anti-TGF-β1 antibody (a-TGF-β, 2 mg/ml) or mouse IgG (mIgG) for 48 h. (D) Cells in which USP3 was knocked down or transfected with scr siRNA were treated with g-TGF-β1 for an additional 48 h. The expression of USP3, E-cadherin and vimentin was detected by Western blot. (E) The morphology of GC cells was observed under an inverted microscope. (F) The GC cells were transfected with USP3 siRNA or scr siRNA for 24 h followed by TGF-β1 treatment for 24 h. Representative images and data from a Transwell assay in GC cells. Each bar represents the mean ± SD. ****, P < 0.001, compared with GC cells treated with TGF-β1. ****, P < 0.001, compared with cells transfected with USP3 siRNA and treated with TGF-β1. The error bars represent the mean ± SD from 3 independent experiments. Scale bars, 50 μm in E. (TIF 4576 kb) [file 13046_2019_1270_MOESM3_ESM.tif]
